# Post-operative *Aspergillus *mediastinitis in a man who was immunocompetent: a case report

**DOI:** 10.1186/1752-1947-4-312

**Published:** 2010-09-23

**Authors:** George Dimopoulos, Iraklis Tsangaris, Garyphalia Poulakou, John Panayiotides, George Tsaknis, Stylianos Orfanos, Apostolos Armaganides

**Affiliations:** 1 Second Department of Critical Care Medicine, 'ATTIKON' University Hospital, University of Athens Medical School, Athens Greece; 2 Fourth Department of Internal Medicine, 'ATTIKON' University Hospital, University of Athens Medical School, Athens Greece; 3 Second Department of Pathology, 'ATTIKON' University Hospital, University of Athens Medical School, Athens Greece

## Abstract

**Introduction:**

*Aspergillus *spp. infections mainly affect patients who are immunocompromised, and are extremely rare in immunocompetent individuals.

**Case presentation:**

*Aspergillus *post-operative mediastinitis is considered to be a devastating infection, usually affecting patients undergoing cardiothoracic surgery with specific predisposing factors. We describe the case of an immunocompetent 68-year-old Caucasian man with severe chronic thromboembolic pulmonary hypertension, who underwent pulmonary thromboendarterectomy and developed post-operative mediastinitis due to *Aspergillus flavus*. The environmental control did not reveal the source of *A. flavus *infection and, despite combined antifungal therapy, our patient died as a result of septic shock and multiple organ failure.

**Conclusion:**

*Aspergillus *mediastinitis mainly affects patients after cardiosurgery operations with predisposing factors, and it is unusual in patients who are immunocompetent. The identification of the *Aspergillus *spp. source is often difficult, and there are no guidelines for the administration of pre-emptive therapy in this population of at-risk patients.

## Introduction

*Aspergillus *spp. infections mainly affect patients who are immunocompromised, usually being acquired by inhalation of small airborne spores [1,2]. The diagnosis of infection caused by this filamentous fungus poses many difficulties, mainly due to the lack of a laboratory method able to discriminate colonization from infection. Not surprisingly, the diagnosis is often confirmed by autopsy [1-3]. The difficulty in diagnosis may lead to delayed treatment, which could be associated with the extremely high mortality rates. Post-surgical mediastinitis is defined as an infection involving the structures between the sternum and the esophagus, occurring mainly after cardiothoracic surgery [4-6]. The highest risk for the development of this infection is associated with orthotopic heart transplantation (2.5 to 6 instances per 100 procedures), while the species usually involved are *Staphylococcus *and *Enterobacter *spp. [7]. *Aspergillus *spp. infections have been described post-operatively in this patient population, but *Aspergillus *mediastinitis has been reported in only 11 patients [1]. A recent published study by Jensen *et al*. reported two cases of mediastinitis among seven cases of post-surgical infection, with a mortality of 100% [8].

We describe post-operative *Aspergillus *mediastinitis in our patient, who underwent pulmonary thromboendarterectomy (PEA). We focus on the clinical dilemmas that could be met and cover the early diagnosis, the difficulties associated with detection of the source of *Aspergillus *spp. and the management of our patient.

## Case presentation

A 68-year-old Caucasian man with severe chronic thromboembolic pulmonary hypertension and progressive decline to New York Heart Association (NYHA) functional class IV was admitted to our intensive care unit (ICU) because of respiratory failure and need for mechanical ventilation. His medical history included repeated episodes of deep vein thrombosis and two episodes of pulmonary embolism despite adequate anticoagulant therapy. Upon completion of the pulmonary hypertension investigation, his pulmonary thromboembolic disease was considered non-operable and was treated with bosentan and inhaled iloprost. Following the initiation of mechanical ventilation he was switched to intravenous iloprost and three months later our patient demonstrated a significant hemodynamic improvement, allowing him to undergo PEA. Details on his hemodynamic status and management are described elsewhere [9].

During his hospitalization, our patient had two episodes of ICU-acquired microbial and fungal infections including a catheter related candidaemia due to *Candida albicans *and *Staphylococcus epidermidis *(isolated from the catheter tip of a left internal jugular central venous catheter, treated with caspofungin for 15 days and linezolid 600 mg twice a day for 14 days), and an episode of ventilator-associated pneumonia (VAP) due to *Acinetobacter baumannii *treated according to the detected antimicrobial susceptibilities with imipenem (1 g every six hours) and colistin (3 × 10^6^U every eight hours).

Immediately prior to PEA, our patient was given 1 g of vancomycin as surgical prophylaxis with the induction of anesthesia. During the first post-operative week he was febrile without an apparent site of infection and received antibiotic treatment according to the surveillance cultures. On the eighth post-operative day an exudate drained automatically from the surgical wound; cultures grown in Sabouraud dextrose agar revealed a fast-growing isolate with cottony texture identified as *Aspergillus *spp. Microscopically, a potassium hydroxide mount confirmed the presence of septate and hyaline, and dichotomously branched hyphae. Conidiophores were uncolored producing spherical to ellipsoidal accessory conidia, covering the entire surface of the biseriate species. Further subcultures in potato flake agar (PFA) were prepared in-house and incubated at 35°C, and identified the presence of *Aspergillus flavus*. The isolate grew rapidly, producing olive-green colonies. When examined by tape mounts, a slide culture prepared on PFA cultured isolate revealed globose vesicles up to 20 μm in diameter, a biseriate arrangement with metulae and phialides, and globose conidia (3.0 to 6.0 μm in diameter); all features similar to those produced in Sabouraud dextrose agar and characteristic for *A. flavus*. A computed tomography (CT) scan of his thorax revealed no specific signs of mediastinitis. Serum and bronchoalveolar-lavage galactomannan tests were not performed, as they were not available at the time. No specific signs of mediastinitis were seen either.

**Figure 1:**
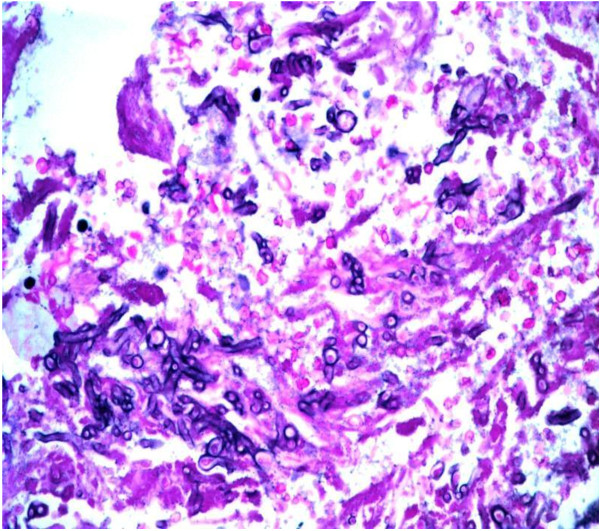
**Fragment of lung tissue with an increased number of hyphae obtained on autopsy (PAS stain ×100)**.

**Figure 2:**
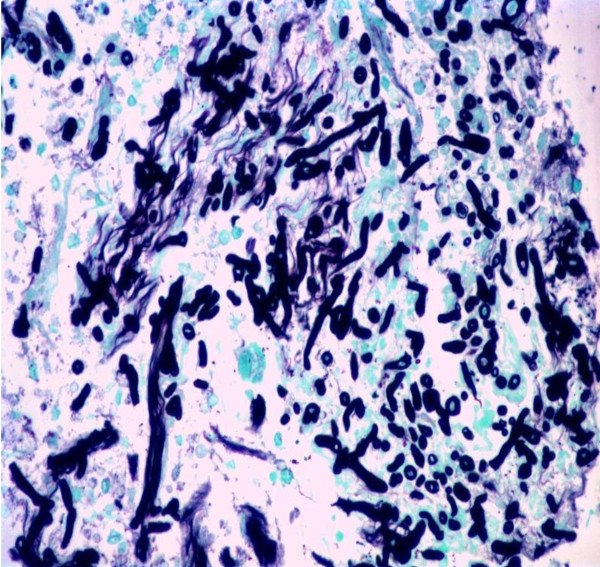
**Fragment of myocardium with hyphae obtained on autopsy (Grocott stain ×100)**.

A combination of liposomal amphotericin B at a dose of 3 mg/kg daily and voriconazole (6 mg/kg twice a day for the first day followed by 4 mg/kg twice a day thereafter) were added because of the severity of the infection, and surgical debridement of the wound was performed. Despite all the therapeutic measures, the results of culture tests remained positive for *A. flavus *(surgical wound necrotic tissue test results were positive for hyphae on direct microscopy after the addition of KOH) and our patient died on the 26th post-operative day because of severe septic shock. Our patient underwent a whole body autopsy. Samples from the sternum, lungs and myocardial tissue fixed in a 10% buffered formol solution were embedded in paraffin; sections 4 μm thick stained with hematoxylin and eosin, PAS and Grocott's silver impregnation stains confirmed the presence of hyphae with morphologic characters pertaining to the genus *Aspergillus *[10] (Figures 1 and 2).

A detailed investigation of all patients undergoing surgical operation during the same period was performed. A total of 50 environmental samples were collected from various sites in the operating room. Four plates were left open for one hour, one of them on the surgical table and the other at various sites on the floor, focusing on the air conditioning system vents. Various surfaces were also sampled with cotton-tip swabs. The filters of the air conditioning system in the surgical room were replaced and samples culture tested. The central supply rooms that housed equipment used in cardiac surgery operations were all inspected for visible signs of mold contamination or damage that might lead to mold growth. Routine scheduled maintenance and replacement protocols for equipment and air filters were reviewed.

None of the patients undergoing surgical operation during the same period developed infection of the surgical site due to *Aspergillus *spp. and the results of environmental samples tested 17 days later were negative. In two of the tested samples hyphomycetes classified as *Cladodosporium *spp. grew on the 17th day, however these were considered as laboratory contamination. These positive samples derived from a storage area in the surgical room. No further isolation of fungi was noted from the plates, which were kept for one month after the environmental sampling inoculation. Additionally, a subsequent investigation was performed on the ICU staff using nasal cultures for possible nasal colonization with *Aspergillus *spp., also giving negative culture test results.

## Discussion

*Aspergillus *mediastinitis has been defined by the US Centers for Disease Control and Prevention as the presence of a positive culture result from a mediastinal sample plus one of the following: (a) fever of over 38°C, or (b) chest pain or sternal instability with purulent effusion in the mediastinum or positive culture results from surgically obtained samples or blood. In a recently published review Pasqualotto *et al*. summarized cases of *Aspergillus *mediastinitis reported in the literature, all in patients who had undergone surgery (four with deep sternal wound infections, two with heart transplantation, two with aortitis, one with patch infection after repaired Fallot tetralogy and two patients who were immunocompetent who were affected during an outbreak of aspergillosis related to a contaminated ventilation system in the operating room). The authors considered immunocompromised individuals with defects in alveolar macrophage and neutrophil function as patients at risk (that is, patients undergoing chemotherapy, bone marrow recipients, solid-organ transplants, or patients with congenital or acquired immune disorders). The automatically drained exudate from the surgical wound in our patient, who was feverish, supported the diagnosis of mediastinitis, despite the absence of confirmatory signs from his thorax CT scan. However our patient was low risk for *Aspergillus *spp. infection development, since he did not fulfill the traditional criteria of immunosuppression (malignancy, chemotherapy or radiotherapy, neutropenia, systematic or immunosuppressive diseases, or systematic steroid treatment). Although our patient had received a short course (7 days) of treatment with steroids (50 mg twice a day) during a septic episode, it is highly unlikely that this resulted in a permanent altered host response. According to Pasqualotto *et al*. the most important and crucial point for the diagnosis of *Aspergillus *mediastinitis in patients undergoing cardiac surgery with destructive wound infections and samples that give negative culture test results is a high clinical suspicion [1]. A potential immunomodulatory factor in our case could be the prolonged prostacyclin use. Recent experimental data support an immunosuppressive role for prostacyclin, through impaired regulation of phagocytosis, bacterial killing, and inflammatory mediator production, but the extrapolation of these findings to the clinical setting remains highly speculative [11].

Environmental investigations did not reveal the source of contamination in our patient's case. Despite the lack of an air sampler, a thorough investigation was performed without identification an environmental source. This finding argues against an ongoing source of contamination and is in line with the absence of new cases of infections with *Aspergillus *among patients undergoing surgery within our hospital. Nevertheless, a point source not yet identified cannot be excluded. However, based on previous reports of outbreaks through the ventilation system of the operating theater, the air conditioning filters of the cardiac surgery room were replaced immediately after the identification of the infection [12]. No renovation or construction procedures were being performed at our hospital during this time period. Direct inoculation from our patient's flora could be a possible source, but the common practice of our ICU department consists of testing of bronchial cultures (biweekly) as well as urine and stool cultures (weekly), and this would have revealed such a previous colonization. Pre-existing colonization of bronchial secretions with *Aspergillus *spp. and chronic lung disease have been demonstrated as risk factors in a case-control study of sternal wound infections, but this was not the case for our patient [13]. To the best of our knowledge, there is no recommendation issued for prophylaxis against *Aspergillus *infections in immunocompetent hosts [14]. Interestingly, our patient had received a 15-day caspofungin course as pre-emptive treatment for invasive candidiasis based on colonization of urine and bronchial cultures by *Candida *spp. in conjunction to additional risk factors (prolonged ICU stay, administration of broad spectrum antibiotics, central line placement, mechanical ventilation and total parenteral nutrition administration) [15,16]. The treatment course was terminated 10 days pre-operatively and no selection of fungi resistant to caspofungin was seen on routine surveillance cultures.

## Conclusion

*Aspergillus *mediastinitis mainly affects patients undergoing cardiosurgery operations with predisposing factors, and is unusual in patients who are immunocompetent. The identification of the source of *Aspergillus *spp. is often difficult, and there are no guidelines for the administration of pre-emptive therapy in this population. Our patient could be added to the series of Pasqualotto and Denning as a third immunocompetent patient with post-operative *Aspergillus *spp. mediastinitis [1].

## Consent

Written informed consent was obtained from the patient for publication of this case report and any accompanying images. A copy of the written consent is available for review by the Editor-in-Chief of this journal.

## Competing interests

The authors declare that they have no competing interests.

## Authors' contributions

GD, IT, GT and GP analyzed and interpreted data from our patient with regard to ICU admission, surgical procedure and microbiology. JCP performed the histological examination of the myocardium and the lung, and SO and AA were major contributors to the writing of the manuscript. All authors read and approved the final manuscript.
